# Quantitative bioanalysis of strontium in human serum by inductively coupled plasma-mass spectrometry

**DOI:** 10.4155/fso.15.76

**Published:** 2015-09-29

**Authors:** Srikanth Somarouthu, Jayoung Ohh, Jonathan Shaked, Robert L Cunico, Gerald Yakatan, Suzana Corritori, Joe Tami, Erik D Foehr

**Affiliations:** 1 Pacific BioLabs, 551 Linus Pauling Drive, Hercules, CA 94547, USA; 2 Cosmederm BioScience, 8910 University Center Lane #450, San Diego, CA 92122, USA; 3 Therapeutics Inc., 9025 Balboa Ave., Suite 100, San Diego, CA 92123, USA

**Keywords:** inductively coupled plasma mass spectrometry, method validation, serum, strontium, Yttrium

## Abstract

**Aim:**

A bioanalytical method using inductively-coupled plasma-mass spectrometry to measure endogenous levels of strontium in human serum was developed and validated.

**Results & methodology:**

This article details the experimental procedures used for the method development and validation thus demonstrating the application of the inductively-coupled plasma-mass spectrometry method for quantification of strontium in human serum samples. The assay was validated for specificity, linearity, accuracy, precision, recovery and stability. Significant endogenous levels of strontium are present in human serum samples ranging from 19 to 96 ng/ml with a mean of 34.6 ± 15.2 ng/ml (SD).

**Discussion & conclusion:**

Calibration procedures and sample pretreatment were simplified for high throughput analysis. The validation demonstrates that the method was sensitive, selective for quantification of strontium (^88^Sr) and is suitable for routine clinical testing of strontium in human serum samples.

## Abstract (lay abstract)

**Lay abstract:** The study of strontium in human serum is useful to understand its biological function. Strontium is found naturally in the environment including drinking water and food. This alkali earth metal plays a biological role in bone and has been shown to have therapeutic properties for osteoporosis and to suppress chemically induced sensory irritation and inflammation. A technique called inductively coupled plasma mass spectrometry was used for its superior ability to measure low levels of strontium in biological matrices. Strontium was measured in normal human samples to demonstrate the method utility and correlate with age, gender and race.

Strontium is an alkali earth metal and has chemical properties similar to its neighboring elements calcium and barium. Strontium metal is found naturally and exists in the +2 oxidation state in four stable isotopic forms (^88^Sr at 82.6%, ^86^Sr at 9.9%, ^87^Sr at 7.0% and ^84^Sr at 0.6%) [1]. Strontium is found naturally in the environment including drinking water and food. It is used industrially to produce colored television tubes and to add color to ceramics, glass and fireworks.

Strontium which resembles and belongs to same group as calcium has an unknown positive effects on human health [2]. Experimental data from recent studies support the biological role of strontium as a therapeutic agent for osteoporosis at low doses [3]. Generally, strontium has low toxicological properties [4]. However, some studies suggest interference of excess absorbed strontium with bone mineralization in developing skeleton [5]. Exposure to elevated levels of strontium during bone development has demonstrated toxicity. In a region with high prevalence of rickets, a correlation was seen with strontium rich soil and vegetable nutrition. Increased incidence of rickets was observed in children living in villages having more than 350 mg/kg soil strontium content [6]. The estimated daily intake of strontium by adult humans is about 4 mg strontium per day in many parts of the world [5]. The exposure varies depending on region, dietary intake and environmental factors. By nature of its similarity to calcium, 99% of strontium is found in skeleton. Generally, strontium has low toxicological properties [4]. A number of topical products commonly classified as cosmetics such as creams, gels and lotions contain strontium salts (strontium nitrate and strontium chloride hexahydrate) in their formulation. Topical application of strontium salts has been shown to suppress chemically-induced sensory irritation and inflammation in humans [7].

Several methods to quantitate strontium in biological samples have been described [5]. In nearly all cases, the biological matrix (serum, plasma, tissue, bone, etc.) is treated with acid or base to digest the samples and solubilize the strontium. Serum samples have been diluted with a Triton X-100- HNO_3_ mixture, urine samples diluted with HNO_3_, soft tissues dissolved in a TMAH (Tetramethylammonium hydroxide) solution and bone samples digested with concentrated HNO_3_ [8]. Various analytical methods are available such as neutron activation analysis and atomic absorption spectrometry to determine the levels of strontium in serum, packed blood cells, urine, bone and soft tissues [8–10]. Graphite Furnace Atomic Absorption Spectroscopy (GFAAS), Inductively Coupled Atomic Emission Spectroscopy (ICP-AES) and inductively coupled plasma mass spectrometry (ICP-MS) have all demonstrated utility and sensitivity. However, the detection limits reported are in low µg/ml concentrations, sample preparation is not simple and a detailed systematic validation of a method has not been reported [11–13]. Sensitivity (30 ng/ml LLOQ) of a recently reported method by Zhang *et al*., limited the applicability of the assay to strontium levels above 30 ng/ml [14]. In some cases, the endogenous levels of strontium in serum were lower than 30 ng/ml and lower range of the assay needs to be extended. A sensitive ICP-MS method to measure endogenous and therapeutic levels of strontium in human serum was thus developed and validated. The current method provides a LLOQ of 10 ng/ml, capable of measuring trace levels of strontium in serum samples accurately with simplified sample pretreatment.

A thorough study for quantification of strontium levels in human serum, using sophisticated analytical instruments and validated procedures is essential for biological monitoring of strontium in humans. The following ICP-MS method was developed and validated with a wide quantitative range and appropriate performance characteristics to measure strontium in samples obtained from healthy individuals. Because strontium is relatively common in nature and is readily taken into the body, it was necessary to establish a baseline or average level of strontium in the human population studied. A panel of normal human serum samples derived from whole blood samples obtained from 18 healthy donors was studied to determine the endogenous concentrations of strontium in males and females of various age groups.

## Materials & methods

### Reference materials, chemicals & apparatus

External calibration standards were prepared from 1000 ± 5 µg/ml strontium (Sr) certified reference material (Inorganic Ventures, Lot#F2-SR02036). Yttrium (Y) internal standard was prepared from 100 ± 0.49 µg/ml Yttrium (Y) certified reference material (Inorganic Ventures, Lot#G2-Y02006). Trace metal grade nitric acid from Fisher Scientific (67 to 70% w/w, Lot#1113050) was used to acidify samples. Trace metal grade Tetramethyl Ammonium Hydroxide solution (TMAH, 25% in H_2_O) from Fluka Analytical (Lot#BCBL4972V) and Trace metal grade Ethylenediaminetetraacetic acid (EDTA) from Sigma (99.995% pure, Lot# MKBK5436V) were used to prepare rinse solutions. ICP-MS Stock Tuning Solution and 7500 Series Pulse/Analog factor tuning solutions obtained from Agilent Technologies were used for the performance check of the ICP-MS instrument. Unfiltered Pooled Human Serum (Lot#BRH774400) and individual pooled human serum samples (Lot# BRH774402- BRH774407) were obtained from Bioreclamation. ‘Seronorm^TM^ Trace Elements Serum L-2’ freeze dried reference serum material was obtained from Fisher Scientific (Lot#0903107). Samples were prepared directly in polypropylene autosampler vials from Qorpak (cat. no. 249191). All dilutions were made with freshly collected pure Millipore water that was generated with Milli-Q water purification system (∼18 MΩ/cm). For reagent preparation, 50 ml polypropylene graduated centrifuge tubes with flat caps were used. All tubes (except autosampler tubes) were soaked overnight in 10% HNO_3_ and rinsed with deionized Milli-Q water prior to use.

### Instrumentation & conditions

An Agilent 7700_X_ ICP-MS instrument (Model#G3281A) with an octopole-based collision/reaction cell was used for all measurements. The instrument was equipped with an 89 samples Tray-D type Agilent Integrated Autosampler (I-AS) and Mass Hunter version A.01.02 software. Sample introduction system for the Agilent 7700_X_ ICP-MS instrument consisted of Tygon peristaltic pump tubing (1.02 mm ID), a glass concentric pneumatic nebulizer, a double-pass scott-type spray chamber, a standard quartz torch (2.5 mm ID), a Nickel (Ni) plated sampling cone and a Platinum (Pt) skimmer cone. Argon (99.99%) and Helium (99.99%) from Atlas (CA, USA) were used as plasma and collision gases, respectively. Instrument parameters presented in Table 1 were used for all the validation studies and sample analyses.

### System suitability test of instrument

At the beginning of the each run, a performance check of the ICP-MS instrument was done by executing the hardware settings start-up tests (Torch Axis, EM, Plasma Correction, Standard Lenses Tune, Resolution/Axis, Performance Report) using Agilent tuning solution (1 ng/ml Li, Co, Y, Ce, Tl). Agilent 7500 series P/A (Pulse/Analog) tuning 1 and P/A tuning 2 solutions were used for performing the P/A factor test. A tune report was generated prior to each run using actual batch conditions specified in Table 1 to evaluate performance parameters such as resolution, mass calibration, oxide and doubly charged ratios. Hardware performance reports and batch tuning reports generated before each validation run demonstrated that interferences from oxide and doubly charged ions of cerium were very low (CeO/Ce <1.5%, and Ce^+2^/Ce^+1^ <3%). These lower oxide and doubly charged ratios of cerium in tuning stage specifies optimized conditions of the system (feasible to minimize polyatomic-based interferences formed by other analytes). Mass calibration results were found to be within 0.1 amu (^7^Li, ^89^Y, ^205^Tl) from the true values and full width for each peak at 10% peak height was verified to be less than 0.9 amu as a mass resolution check.

### Calibration standards, quality controls & samples preparation

Ideally calibration standards and quality control samples should be prepared in the matrix (serum) used for study samples. However, the endogenous strontium was at levels significant enough to justify the evaluation of an artificial matrix and other options for calibration standards. An attempt was made to use synthetic serum (UTAK Laboratories Inc., Lot# A0878) as a calibration matrix, however a significant amount of strontium (10 ng/ml) was observed. Therefore, calibration standards were prepared in 1%HNO_3_ (v/v) free of detectable strontium instead of serum or artificial serum. An intermediate stock reference standard (10,000 ng/ml) of strontium was prepared by spiking 10 μl of 1000 µg/ml standard into 990 μl of 1% HNO_3_. This 10,000 ng/ml intermediate stock reference standard was used in the preparation of quality controls (mid and high QCs) and for the preparation of 1000 ng/ml intermediate calibration standard. The 1000 ng/ml intermediate calibration standard was used in the preparation of seven working calibration standards ranging from 10 to 250 ng/ml in 1% HNO_3_.

QC samples at low, mid and high concentrations were prepared in normal unfiltered pooled human serum from Bioreclamation (Lot#BRH774400). Because of the significant endogenous level of the analyte in the human serum, normal pooled human serum without addition of strontium was used as low QC (unspiked). Mid and high QC samples were prepared by spiking the low QC with 80 and 150 ng/ml strontium, respectively. QC sample preparation and expected concentrations are summarized in Table 2.

Yttrium (Y) was used as an internal standard (IS) since its endogenous concentration in human serum is at significantly low level and its mass and ionization potential are near to the analyte (Sr). Internal standard solution was prepared at 100 ng/ml by spiking 200 μl of 100 µg/ml Yttrium standard into 200 ml of 1% HNO_3_. Working calibration standards (10 to 250 ng/ml), QCs (low, mid and high) and study samples were diluted 1 in 10 with 100 ng/ml Yttrium IS (100 ng/ml Yttrium in 1% HNO_3_). For example, 200 μl of each sample was combined with 1800 μl of IS, capping and mixing gently. Approximately a 0.6 to 0.7 ml portion of the diluted sample was aspirated by the ICP-MS with the instrument settings mentioned in Table 1.

Calibration blank and blank samples were prepared by a 1 in 10 dilution of 1% HNO_3_ with IS solution. Rinse Solution (4% v/v TMAH/0.03% w/v EDTA) was prepared using Milli-Q water as diluent.

This ICP-MS method has been developed and validated in accordance with the US FDA Guidance for Industry, Bioanalytical Method Validation, US Department of Health and Human Services, Food and Drug Administration, Center for Drug Evaluation and Research (CDER), Center for Veterinary Medicine (CVM), September 2013 Biopharmaceutics. As the objective of the method validation is to guarantee the quality of the results of the authentic analyte in the natural biological matrix, actual validation samples were aliquots of the authentic matrix.

### Experimental design

Clear homogenous solutions were obtained when serum samples were diluted in 1% HNO_3_ (v/v). When diluted with a higher concentration of nitric acid (≥2% HNO_3_), significant precipitation of organic matter was observed. Using 1% HNO_3_ (v/v) to dilute serum samples to measure strontium by ICP-MS and Neutron Activation Analysis was already demonstrated in previous studies [1]. Dilution of the serum sample is necessary as it reduces the blockage of sample and skimmer cone orifices [1]. Even after longer sequences (∼100 samples) of these diluted serum samples, no clogs were observed and sample transportation was not obstructed. To achieve steady internal standard signal intensity, internal standard (yttrium) was added to the diluent. Matrix matched calibration techniques (such as method of standard additions, isotope dilutions, addition calibration, isotope dilution, surrogate matrix and surrogate analyte approaches) were avoided due to potential complications to the validation procedure and being labor intensive when analyzing large number of samples. Samples were calibrated against nonmatrix matched external calibration standards (injected at the beginning of the sequence) and no recalibration was performed. Calibration standards were prepared in 1% HNO_3_, as it was not workable to construct a calibration curve in specific serum matrix, due to small but significant differences in the endogenous levels of strontium in individual serum samples are not being compensated. To minimize carryover of analytes, a rinse solution comprising 4% TMAH/0.03% EDTA solution was injected after calibration standards, QCs and in between the samples. Carryover was assessed by injecting blank samples after rinse solutions. Memory effects were significantly minimized, as no apparent systematic drift was observed and analyte response in the blanks in all the runs was observed to be ≤3 ng/ml. Trace elements tubes (Royal Blue-Becton Dickinson) were used for serum separation to minimize contamination. Collected serum after extraction was transferred to cryovials free of trace elements. Final working calibration standards, QCs and sample dilutions were performed in autosampler vials directly to prevent loss of strontium and to prevent contamination.

Ion lens voltages were optimized manually by monitoring analyte signal at mass 88 during tuning procedure to achieve maximum sensitivity. Helium as a collision gas was introduced at a lower flow rate (1.5 ml/min) which improved the sensitivity and quality of the analytical signal. Helium at a higher flow rate suppressed the analyte signal, which ultimately affected precision in analyte peak quantification at lower analyte concentrations. Helium at a lower flow rate for this application is acceptable, as there are no common interfering elements for ^88^Sr present in the serum matrix.

## Results

### Method validation

The method was extensively evaluated covering all important performance characteristics such as specificity, linearity, accuracy, dilution linearity, precision and stability.

### Specificity

Method specificity is defined as the ability of an analytical method to differentiate and quantify the analyte in the presence of other components in the sample matrix. Due to the endogenous level of the analyte (Sr) in the human serum, specificity of the current method was demonstrated by analyzing six different individuals of human serum samples (from Bioreclamation). These six lots of serum samples were processed without internal standard (double blank serum samples, one in ten dilution with 1%HNO_3_), with internal standard (blank serum samples, one in ten dilution with internal standard solution), and strontium (Sr) spiked into six different individual lots at 50 ng/ml (further diluted one in ten with internal standard solution). Results from specificity experiment are presented in Table 3.

Acceptable mean accuracy (98%) was obtained for all the six matrix types, when fortified at 50 ng/ml strontium. No significant response was observed for internal standard (yttrium) in all the six lots. In addition to this, the isotope selected (^88^Sr) for monitoring, is free from significant interferences. Other isotopes of strontium (^84^Sr, ^86^ Sr, ^87^Sr) are subject to significant isobaric spectral interference from krypton (^84^Kr, ^86^Kr present as impurity in Argon) and rubidium (^87^Rb from serum) [1]. In dilution linearity experiments, measured concentrations of a fivefold diluted serum samples (n = 3) were in good agreement (mean accuracy >98%) with original undiluted serum samples. This indicates that there are no significant matrix effects by the presence of other easily ionizable elements. So, it can be concluded that the method was selective for strontium (^88^Sr).

### Calibration curve

A calibration standard curve is the relationship between the instrument response (analyte cps/internal standard cps) and the known concentration of the analyte (in ng/ml). A calibration curve is used to demonstrate linearity, the ability of a method to elicit test results proportional to the concentration of the analytes within a given range. Since human serum contains endogenous strontium, calibration standards were prepared in a diluent/internal standard solution containing 1% HNO_3_ and 100 ng/ml Yttrium (IS). Calibration blank sample and seven nonzero calibration standards of analyte (Sr) with concentrations 10 ng/ml, 25 ng/ml, 50 ng/ml, 100 ng/ml, 150 ng/ml, 200 ng/ml and 250 ng/ml were injected at the beginning of each run. For all the validation experiments, a linear regression curve with no weighting was used to calibrate the instrument response. All calibration curves had correlation coefficient (R) of ≥0.999. Signal to noise ratio, calculated as the ratio of analyte response (counts per second [cps]) at LLOQ (10 ng/ml) over blank sample response is ≥5 in all the runs. Accuracy of back calculated concentrations for calibration standards was within 10% of nominal value (except for LLOQ, which within 15% of nominal value).

### Accuracy & method precision

Precision is the repeatability or closeness of agreements between a series of measurements for a statistically significant number of samples (multiple sampling of the same homogenous sample). The accuracy of an analytical method describes the closeness of agreement between the test results obtained by a method to the conventional true value or an accepted reference value.

To determine accuracy and method precision, five sets of QC samples over three concentration levels (LQC, MQC and HQC) covering the specified range were prepared and analyzed. Accuracy and method precision were calculated for five injections of LQC (unspiked), MQC (80 ng/ml spike) and HQC (150 ng/ml spike) samples. Accuracy and method precision results obtained are presented in Table 4.

### Dilution linearity & method precision of >ULOQ samples

During the analysis of samples, if any sample result was higher than the Upper Limit of Quantitation (ULOQ) range, the sample had to be diluted with 1% HNO_3_ to achieve a result (concentration) within the calibration range. To accommodate analyzing samples originally 2× above the ULOQ (250 ng/ml), recovery for diluted serum samples was validated at approximately 100 ng/ml. A 500 ng/ml spiked serum sample was prepared, fivefold diluted with a 1% HNO_3_ and analyzed according to the method (n = 3). The mean accuracy of diluted samples (n = 3) was within 2% of undiluted values and relative standard deviations (%RSDs) were within 0.1% (n = 3). These dilution linearity (fivefold) results obtained also indicated that there were no significant matrix effects.

### Precision

Repeatability was determined by preparing multiple aliquots from a single homogenous lot of serum. Intraday precision is a measure of within run repeatability (typically less than 24 h), determined by injection of six replicates at each QC level (LQC, MQC and HQC). Precision results obtained are presented in Table 5.

Interday precision is a measure of the repeatability of the concentration measurement from day-to-day and from assay-to-assay. Interday precision was determined by injection of three replicates at each QC level on three separate days (day 1, 2 and 3). The day 2 analysis was performed 43 h after day 1 (t = 0) and the day 3 analysis was performed 67 h after day 1. Aliquots of LQC (unspiked), MQC (80 ng/ml spike) and HQC (150 ng/ml spike) samples were stored at -20°C, 3 aliquots of each sample were prepared (diluted with internal standard) prior to analysis and analyzed according to the method (n = 3) on three separate days against a freshly prepared standard curve.

### Stability

Stability of strontium in human serum was evaluated at ambient temperature storage, frozen storage and through three freeze–thaw cycles. Stability of the prepared standards and samples (diluted with internal standard) was determined at ambient temperature.

### Postpreparative stability

Postpreparative stability was performed to define the period of time in which the prepared samples (calibration standards, LQC, MQC and HQC samples) are stable and representative of freshly prepared samples. Stability was evaluated by comparing the results from stored samples (for ≥24 h and ∼3 days at ambient temperature with caps closed) versus freshly prepared samples. Two replicates of standards (25, 100 and 200 ng/ml) and nine replicates of samples (at each QC level) were prepared (one in ten diluted with internal standard). Duplicate injections of each standard were made at 43 h and at approximately 3 days (Table 7) against freshly prepared standards. Three replicates of LQC, MQC and HQC samples were injected in duplicate as t = 0 along with freshly prepared standards. The remaining six replicates of samples were stored at ambient temperature. Three replicates of samples were injected in duplicate at 43 h and the remaining three replicates were injected at approximately 3 days against freshly prepared standards. Postpreparative stability results obtained are presented in Table 6A & Table 7.

### Prepreparative stability and long-term stability

Stability of the analyte in the matrix was evaluated at ambient temperature (bench top stability) and storage stability at ≤-20°C was performed. Short-term stability of the samples was evaluated for 43 h at ambient temperature. Long-term sample stability was assessed by storing samples at ≤-20°C for approximately 1 week and 2 months. Aliquots of LQC (unspiked), MQC (80 ng/ml spike) and HQC (150 ng/ml spike) samples without IS addition were stored at ambient temperature and at ≤-20°C. Prior to analyses, samples were diluted with IS and analyzed according to the method (n = 3) against a freshly prepared standard curve. Prepreparative stability results obtained are presented in Table 8.

### freeze–thaw stability

Stability of the analyte in the matrix was evaluated through three freeze–thaw cycles by freezing samples at ≤-80°C. Aliquots of LQC (unspiked), MQC (80 ng/ml spike) and HQC (150 ng/ml spike) samples without IS addition were stored at ≤-80°C. After three freeze–thaw cycles, samples were diluted with IS and analyzed according to the method (n = 3) against a freshly prepared standard curve. The mean accuracy (% of t = 0) after three freeze thaw cycles were within 5% of expected values (102.9% for LQC, 104.2% for MQC and 104.8% for HQC samples).

## Measurement of strontium levels in normal human population

To verify the validity of the calibration curve and to monitor system performance throughout the analytical run, one set of QCs were injected once in every 20 study samples. For QC accuracy calculations of each assay, the measured low QC value (average of duplicate injections of low QCs before the injection of study samples) was used as the expected low QC concentration for the assay. For all analyses, correlation coefficients (R) of calibration curves were equal to or higher than 0.9999 and accuracy of bracketing QCs ranged from 90 to 105% of each nominal concentration.

Analytical results of endogenous strontium levels in 24 serum samples (six samples from Bioreclamation) prepared from individual human subjects are presented in Table 9. Major demographic characteristics of subjects participated in the study were also reported.

## Discussion

Based on the data presented, the bioanalytical ICP-MS method developed is accurate, sensitive, precise and selective for quantification of strontium (^88^Sr) in human serum. The available stability data indicate that postprocessed serum samples (diluted with internal standard solutions) are stable at ambient temperature for 3 days. Preprocessed serum samples (undiluted) are stable for at least three freeze–thaw cycles, stable for 2 days (at ambient temperature) and 2 months at ≤-20°C. The validation demonstrated that this method can be used in support of routine clinical testing of strontium (^88^Sr) in human serum samples. Consideration of spectral and nonspectral interferences is essential to determine elements in biological matrices. A number of techniques can be employed to eliminate interferences such as calibration procedures, internal standardization, sample dilution, mathematical correction equation, chemical separation and matrix modification [15]. In the present experimental design, interferences are effectively eliminated by using simple proven approaches such as internal standardization, specimen dilution, choice of isotope and fine-tuned instrumental conditions. The one in ten dilution of the serum sample (with internal standard solution) reduced the physical suppression of the analyte signal by the dissolved solids in the sample and resulted in excellent spike recoveries. Yttrium served as an ideal internal standard by correcting matrix-induced signal fluctuation and reducing the long-term signal drift. Yttrium behaves in similar manner to strontium in plasma, because of its nearest mass (89 amu) and ionization potential (600 KJ/mol) to that of strontium mass (88 amu) and ionization potential (550 KJ/mol). Possible polyatomic interfering species for strontium (Sr^88^) are calcium dimers (^44^Ca_2_, ^42^Ca^46^Ca, ^40^Ca^48^Ca), calcium argides (^48^Ca^40^Ar), iron oxides (^56^Fe^16^O_2_), zinc oxides (^70^Zn^18^O) germanium oxides (^72^Ge^16^O) and doubly charged species of ^176^Yb, ^176^Lu, ^176^Hf at significantly higher levels [16,17]. None of these interferences are expected given these elements are at lower concentrations and rare and oxide formation was shown to be minimal. Results obtained confirm that Sr^88^ is free from interferences at this low levels in serum and is recommended for routine clinical analyses. When analyzing Sr^88^ in high calcium, iron and zinc containing samples, use of interference correction equations combined with optimized instrumental parameters such as sampling depth, ion lens settings, nebulizer and collision gas flow is recommended. However, if the intensity of analyte signal is extremely low and intensity of interfering ions is significantly high, use of mathematical correction equation is not suitable [18]. Alternate approaches such as cool plasma conditions, matrix precipitation, matrix modification, analyte preconcentration techniques have to be considered to compensate for spectral interferences.

Statistically similar type of experimental results were obtained by Zhang *et al*. [14] in a recent method validation study to measure strontium levels in serum using ICP-MS. A mean value of 50.5 ± 6.4 ng/ml (n = 12, healthy male chine subjects) strontium in human serum reported by Zhang *et al*., fall within the reference range (19 to 96 ng/ml) reported in this study. In comparison of both experimental approaches, following differences appear to be particularly interesting. Zhang *et al*. used 7:93 (V/V) ratio of hydrogen/helium mixed gas as a collision gas at 3.75 ml/min. This current work demonstrates that helium gas mode alone at lower flow rate (1 ml/min) is sufficient for ^88^Sr analysis in serum. Analysis time is shorter in single gas mode analysis when compared with multiple mode analysis as longer stabilization delays are not necessary. A calibration curve ranging from 30 to 15,000 ng/ml (LLOQ set to 30 ng/ml) used in Zhang *et al*. method is applicable to quantitate strontium over a wide range of concentrations in clinical study samples. But to measure trace levels of strontium reliably, a high sensitive assay is required. Lower limit of quantitation established at 10 ng/ml in the present method is accurate enough to measure trace levels of strontium in serum and is reproducible with acceptable signal to noise ratio. On the other hand, current method was validated to evaluate dilution linearity, to dilute and analyze test samples with strontium levels exceeding the ULOQ. We conclude that our approach offers advantages from analytical and economical point of view for high throughput analysis. Signal to noise ratio at LLOQ can be improved significantly by reducing background contribution from the system using high purity reagents, decontaminated containers and clean operating conditions of laboratory. Limits of detection can be improved by further optimization of method, but achieving quantitation limits less than 10 ng/ml may not be important for this study as it is outside the physiologically relevant range. With minor optimization of the experimental conditions, demonstrated ICP-MS method can be tailored to detect and quantify other physiological elements present in serum at lower concentrations such as rubidium, boron, copper, zinc and aluminum. To apply the current method for routine multielemental analyses, fine tuning of instrument to obtain optimum signal intensity for intended mass range is needed. Spectral interferences associated with either the plasma, or matrix components of sample/solvent have to be compensated. Using element specific internal standards (of close mass and ionization potential to that of analytes) across entire mass range of analytes is essential to minimize matrix effects. However, method detection limits will be compromised as obviously single-element monitoring yields better detection limits than multielemental analyses.

A mean value of 34.6 ± 15.2 ng/ml (n = 24) found in this study is in close agreement, but slightly lower than the previously published mean value of 49.2 ± 172 ng/ml (n = 110) by Forrer *et al*. [19]. The observed differences in the ranges may be because of uneven distribution of strontium in geographical regions, dietary intake, age, sex and environmental factors. This explanation is supported by previous observation that trace elements concentrations may reflect age, sex and social status of individual [20]. Statistical evaluation of the levels of strontium by age, gender, diet, race and region will help to better understand the global distribution pattern.

## Future perspective

Proposed methodology can be enhanced for simultaneous multielemental analyses in serum by further optimization of experimental conditions and data collection parameters. High concentrations of inorganic constitutes in clinical samples trigger matrix and spectral interference problems, hence achieving best detection limits for physiological elements (especially with masses from 47 to 82) which, at ultratrace levels, can be challenging. This demands a highly sensitive, accurate assay capable of resolving matrix and spectral interferences for high throughput multielemental analyses to support bioanalytical studies. To the best of our knowledge no study has been done to determine racial, gender and geographical variation of strontium levels in human serum. The current study is limited by sample size and endogenous strontium levels determined are insufficient for statistical interpretation. Current method can be employed for further extensive studies to gain insight into strontium distribution and to establish reference ranges respective to age, race and gender.

**Table 1.** **Inductively coupled plasma-mass spectrometry instrument settings.**

ICP-MS instrument	Agilent 7700x -ICP-MS	
Analyte mass	Strontium (Sr) m/z: 88^†^	
IS mass	Yttrium (Y) m/z: 89	
Plasma gas	Argon	
Collision gas	Helium	
Recommended parameters	Plasma gas (l/min)	15
	Helium flow (ml/min)	1.5
	Carrier gas (l/min)	0.45
	Make up gas (l/min)	0.69
	Plasma RF power (W)	1550
	Plasma RF matching (V)	1.70
	Sampling depth (mm)	7.5
	S/C temp (˚C)	2
	Extract 1 lens (V)	1.1
	Extract 2 lens (V)	-200
	Omega bias (V)	-75
	Omega lens (V)	9.8
	Cell entrance (V)	-36
	Cell exit (V)	-60
	Deflect (V)	14
	Plate bias (V)	-35
	Energy discrimination (V)	4.5
	OctP Bias (V)	-8.0
	Oct RF (V)	180
	Acquisition mode	Spectrum
	Peak pattern	1
	Replicates	3
	Sweeps/replicate	1000
	Integration time/mass (s)	0.7

^†^^88^Sr isotope was monitored because other isotopes (^84^Sr, ^86^ Sr, ^87^Sr) are subject to significant isobaric interference from Krypton (^84^Kr, ^86^Kr present as impurity in Argon) and ^87^Rb from serum [1].

**Table 2.** **Preparation of quality controls.**

**QC ID**	**Volumes used (μl)**		**Spiked strontium concentration in serum (ng/ml)**	**Expected strontium concentration in serum (ng/ml)**
	**10,000 ng/ml stock std (μl)**	**Matrix, serum (μl)**		
Low QC	0	1000	0	Observed
Mid QC	8	992	80	80 + low QC result
High QC	15	985	150	150+ low QC result

QC: Quality control.

**Table 3.** **Analyte response for specificity.**

**Source lot #**	**Endogenous analyte (^88^Sr) concentration (ng/ml)**	**Expected analyte (^88^Sr) concentration (endogenous+50 ng/ml spike) (ng/ml)**	**Measured analyte (^88^Sr) concentration (ng/ml)**	**Accuracy (%)**
BRH774402	25	75	74	99
BRH774403	22	72	70	98
BRH774404	51	101	100	99
BRH774405	32	82	81	99
BRH774406	22	72	72	101
BRH774407	44	94	90	96
			Mean %accuracy	98.6
			CV (%)	1.5

**Table 4.** **Accuracy and method precision results (n = 5).**

**Injection #**	**LQC (unspiked) (ng/ml)**	**MQC (80 ng/ml spike) (ng/ml)**	**HQC (150 ng/ml spike) (ng/ml)**
1	40	117	187
2	40	116	182
3	40	118	183
4	43	117	182
5	47	118	185
Mean	42	117	184
%RSD	7.6	0.6	1.2
Mean accuracy (%)	N/A	96.1	95.7

Reference Serum Material: To further ensure the accuracy and validity of the method, freeze dried reference serum material “Seronorm^TM^ Trace Elements Serum L-2 (Lot#0903107)” was analyzed for strontium (^88^Sr) during validation. One vial was reconstituted with 3 ml of Millipore water, dissolved completely by vortexing gently. A value of 35 ng/ml (average of duplicate injections) was obtained, which is within 4% of the certified value (∼36.3 ng/ml by inductively coupled plasma-sector field mass spectrometry).

**Table 5.** **Intraday and interday precision results.**

**Sample type**	**Intraday precision (%RSD) n = 6**	**Interday precision (%RSD) n = 9**			
		**Day 1 (n = 3)**	**Day 2 (n = 3)**	**Day 3 (n = 3)**	**Total (n = 9)**
LQC (unspiked)	1.5	2.0	1.1	0.5	2.9
MQC (80 ng/ml spike)	0.4	0.5	1.1	2.9	3.2
HQC (150 ng/ml spike)	0.4	0.5	0.9	0.6	2.3

**Table 6.** **Postpreparative stability results of samples.**

**Duration**	**Replicate no.**	**LQC (unspiked) (ng/ml)**	**MQC (80 ng/ml spike) (ng/ml)**	**HQC (150 ng/ml spike) (ng/ml)**
Day 1	1	39	119	189
	1	39	118	189
	2	39	118	188
	2	38	118	188
	3	39	118	187
	3	39	118	187
Avg t = 0		**38.7**	**118.2**	**187.7**
Day 2	1	44	122	195
	1	45	122	195
	2	44	127	204
	2	44	127	204
	3	42	127	202
	3	42	127	202
Avg day 2		**43.5**	**125.3**	**200.2**
Day 2 (% of t = 0)		**112.3**	**106.0**	**106.7**
Day 3	1	41	128	197
	1	40	128	198
	2	44	121	196
	2	44	121	196
	3	42	122	199
	3	42	123	198
Avg Day 3		**42.0**	**123.8**	**197.2**
Day 3 (% of t = 0)		**108.5**	**104.7**	**105.1**

**Table 7.** **Postpreparative stability results of standards.**

**Duration**	**Injection no.**	**25 ng/ml std (ng/ml)**	**100 ng/ml std (ng/ml)**	**200 ng/ml std (ng/ml)**
Day 1 (t = 0)		26	100	200
Day 2	1	29	106	212
	2	29	106	212
Avg day 2		29.0	105.9	211.9
Day 2 (% of t = 0)		110	105.9	106.0
Day 3	1	26	104	209
	2	26	104	208
Avg Day 3		26.3	104.2	208.4
Day 3 (% of t = 0)		99.8	104.2	104.3

**Table 8.** **Prepreparative stability results.**

**Duration**	**Temperature**	**Replicate no.**	**LQC (unspiked) (ng/ml)**	**MQC (80 ng/ml spike) (ng/ml)**	**HQC (150 ng/ml spike) (ng/ml)**
Day 1	N/A	1	39	117	186
	N/A	2	37	118	188
	N/A	3	38	118	188
Avg day 1			**38.2**	**117.8**	**187.4**
Day 2	Ambient	1	41	128	201
	Ambient	2	41	127	200
	Ambient	3	41	129	200
Avg day 2			**41.2**	**127.8**	**199.9**
Day 2 (% of t = 0)			**107.9**	**108.4**	**106.7**
1 week	≤-20°C	1	39	121	195
	≤-20°C	2	39	123	198
	≤-20°C	3	40	122	199
Avg 1 week (ng/ml)			**39.1**	**121.7**	**197.3**
1 week (% of t = 0)			**102.4**	**103.3**	**105.3**
2 months	≤-20°C	1	42	129	207
	≤-20°C	2	43	130	207
	≤-20°C	3	43	132	207
Avg 2 months (ng/ml)			**42.7**	**130.6**	**206.8**
2 months (% of t = 0)			**111.6**	**110.9**	**110.3**

**Table 9.** **Endogenous strontium levels observed in healthy human population.**

**Subject**	**Age (years)**	**Gender**	**Race**	**Concentration (ng/ml)**
1	22	Female	Not Hispanic or Latino White	31
2	23	Male	Not Hispanic or Latino White	19
3	22	Female	Hispanic or Latino White	35
4	40	Male	Not Hispanic or Latino White	47
5	27	Male	Not Hispanic or Latino White	27
6	53	Female	Not Hispanic or Latino White	31
7	27	Male	Not Hispanic or Latino White	32
8	25	Male	Not Hispanic or Latino White	38
9	67	Male	Not Hispanic or Latino White	96
10	49	Female	Not Hispanic or Latino White	24
11	32	Female	Hispanic or Latino White	28
12	54	Male	Not Hispanic or Latino Black or African American	42
13	30	Female	Not Hispanic or Latino Multiple White and Black	31
14	27	Male	Not Hispanic or Latino White	36
15	30	Female	Not Hispanic or Latino White	41
16	23	Female	Not Hispanic or Latino White	24
17	24	Male	Not Hispanic or Latino White	24
18	38	Female	Not Hispanic or Latino White	28
19	28	Male	Hispanic	25
20	36	Male	Black	22
21	50	Male	Black	51
22	49	Female	Hispanic	32
23	21	Female	Hispanic	22
24	26	Female	Caucasian	44

**Executive summary**

**Background**

The study of strontium in human serum is useful to understand its biological function. A systematic validation of a sensitive assay capable of measuring strontium in serum at low ng/ml concentrations has not been reported. This report details bioanalytical ICP-MS method validation, developed to measure endogenous and therapeutic levels of strontium in human serum.

**Method/results**

Simplified sample preparation and calibration procedure is well suited for high throughput clinical applications related to study of strontium in human serum. Endogenous levels of strontium present in human serum samples ranged from 19 to 96 ng/ml.

**Discussion/conclusion**

The method is sensitive, accurate, precise and reproducible. The study results indicate that method was successfully validated and applied for analysis of strontium in human serum samples. Results from the current study and previous studies indicate that endogenous levels varied significantly between individuals. Observed variability could possibly be explained by difference in age, sex and other geographical differences.
